# Difficulty in Micturition in Nervous Patients Admitted to Hospital Wards

**Published:** 1908-04-25

**Authors:** 


### Resident Medical Officers' Department.

### DIFFICULTY IN MICTURITION IN NERVOUS PATIENTS ADMITTED TO HOSPITAL WARDS.

It is a common occurrence for nervous patients, when admitted for the first time to a hospital ward, and confined to bed, to have some difficulty in micturating; but this is very frequently overcome after one or two trials, and nothing further is then heard of it. Occasionally, however, it happens that, after repeated trials, in different positions, and in spite of "screens" being placed partly round the bed, a very nervous patient finds himself quite unable to pass water, even in presence of much discomfort and pain from a distended bladder.

In all the cases in which difficulty to this extent has been observed by the writer, the patients had also stammering speech, and this inco-ordination of the muscles of articulation probably has a close connection with the difficulty of co-ordinating those of the bladder and urethra. Only in the greatest privacy can such a patient begin micturition. In such a marked case, it not infrequently happens that a thoughtful ward sister will prepare catheters and lotions in readiness for the house surgeon's visit; but, before a resident proceeds to pass a catheter he should make perfectly sure that the patient has had every chance of emptying his bladder naturally. In all probability the nurse will have placed screens partly round the bed when the patient was given the urinal, but this is not privacy enough for such a subject. If, however, one completely surrounds the bed with screens, and so allows the patient as much privacy as is possible, he will in most cases be able to overcome the difficulty.

In some cases even, it would be wise, if possible, to place the patient in a private side ward, rather than begin using a catheter, for one passage of the latter does not end the difficulty, and the patient may require its use again and again; whereas, once he has managed to co-ordinate his muscles under these conditions, and he becomes accustomed to ward life, the difficulty gradually disappears. The Junior Resident, however, is at times inclined to take advantage at once of the catheters prepared for him rather than give the trouble of removing the patient to a side-room; but, although there is little danger in using a properly prepared catheter, it is a much more scientific, as well as a more tactful, solution of the difficulty to gain the end by natural means.

